# *Kaempferia parviflora* and Its Methoxyflavones: Chemistry and Biological Activities

**DOI:** 10.1155/2018/4057456

**Published:** 2018-12-16

**Authors:** Dalin Chen, Hongliang Li, Wen Li, Shuo Feng, Dingsen Deng

**Affiliations:** ^1^Gannan Health Vocational College, Ganzhou 341000, China; ^2^College of Pharmacy, Gannan Medical University, Ganzhou 341000, China

## Abstract

*Kaempferia parviflora* (KP), a health-promoting herb, has been traditionally used for treating a variety of diseases. Pharmacological studies have claimed the various benefits from KP and its main effective methoxyflavones, including cellular metabolism-regulating activity, anticancer activity, vascular relaxation and cardioprotective activity, sexual enhancing activity, neuroprotective activity, antiallergic, anti-inflammatory, and antioxidative activity, antiosteoarthritis activity, antimicroorganism activity, and transdermal permeable activity. These might be associated with increased mitochondrial functions and activated cGMP-NO signaling pathway. However, the underlying molecular mechanisms of KP and its methoxyflavones are still under investigation. The clinical applications of KP and its methoxyflavones may be limited due to their low bioavailability. But promising strategies are on the way. This review will comprehensively discuss the biological activities of KP and its methoxyflavones.

## 1. Introduction

*Kaempferia parviflora* (KP) or Krachaidam, which belongs to the family Zingiberaceae, is originally found in the North and Northeast of Thailand. The rhizomes of KP, also known as black ginger, are popular as health-promoting herbs and traditionally used as a folk medicine for managing a variety of diseases, including inflammation, ulcers, gout, colic disorder, abscesses, allergy, and osteoarthritis [1, 2]. A number of pharmacological researches on KP have claimed the valuable benefits for a variety of diseases. In this review, we will discuss the activities of methoxyflavones, including cellular metabolism-regulating activity, anticancer activity, vascular relaxation and cardioprotective activity, sexual enhancing activity, neuroprotective activity, antiallergic, anti-inflammatory, and antioxidative activity, antiosteoarthritis activity, antimicroorganism activity, and transdermal permeable activity.

## 2. Chemical Structures, Metabolism, and Toxicology

**Figure 1:**
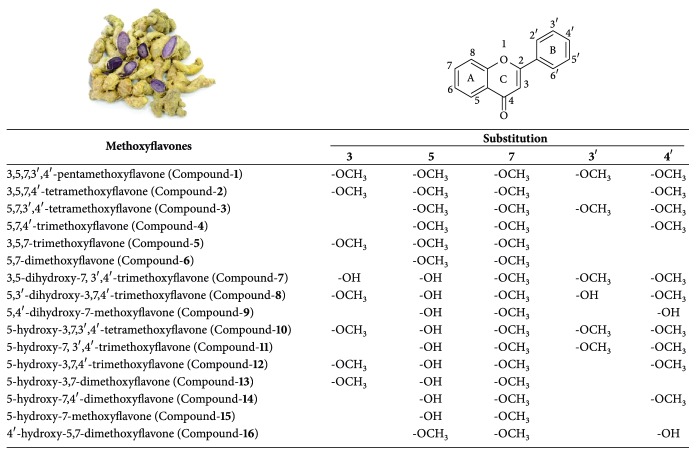
Picture of KP rhizomes (left) and identified structure of methoxyflavones (right) isolated from KP. The main structure of methoxyflavone includes benzene A ring with 2 substituent groups at positions 5 and 7, an aromatic B ring with 2 substituent groups at positions 3′ and 4′, and C ring with a substituent group linking on position 3. The substituent groups might be -H, -OH, or -OCH3.

Phytochemical study of KP has revealed that the main methoxyflavones have been structurally identified [3, 4] (Figure 1). To investigate the antiallergic activity in inhibiting degranulation, structure-activity relationship (SAR) of KP methoxyflavones studies shows that methoxylation at position 5 and vicinal methoxylation at positions 3′ and 4′ play the crucial roles in promoting this activity [5]. 7-Methoxyflavones are included for testing their anticholinesterase activity. Compound**-4** and Compound**-6** have been demonstrated to exhibit highest inhibitory effects on acetylcholinesterase (AChE) and butyrylcholinesterase (BChE) activity. SAR study shows that dimethoxylation at positions 5 and 7 and a free substituent at position 3 are necessary for the inhibitory effects on AChE and BChE. Hydroxylation at position 5 decreases such effects [6]. In another study shows that the methoxyl group at position 5 in 7-methoxyflavones plays a crucial role in PDE-5 inhibition. However, methoxylation at position 4′ shows no benefits for PDEs inhibition [7]. Furthermore, methylation at position 5 reduces the cytotoxicity of methoxyflavones to B16 melanoma 4A5 cells. Thus, the cytotoxicity of Compound**-11** is more than that of Compound**-3 **[8]. Structural modifications of KP methoxyflavones have been investigated. Several oxime derivatives from Compound-**6** are synthesized and found to show cytotoxicity against HepG2 and T47D cell lines [9].

Three methoxyflavones including 3,5,7,3′,4′-pentamethoxyflavone (Compound-**1**), 5,7,4′-trimethoxyflavone (Compound-**4**), and Compound-**6** are selected for investigating the characteristics of pharmacokinetics. They quickly reach their peak concentration within 1-2 h after oral administration in rats. However, they show a very low bioavailability of 1%-4%. The distribution study of methoxyflavones indicates they exist in liver, kidney, lung, testes, and brain. They are metabolized and eliminated through urine after demethylation, sulfation, and glucuronidation [10]. The human intestinal bacteria for metabolizing methoxyflavones are screened through activity-guided assays under anaerobic conditions. Compound-**6** and Compound-**4** are completely transformed into 5,7-dihydroxyflavone (chrysin) and 5,7,4′-trihydroxyflavone (apigenin), respectively. It has been revealed that methoxyl group at position 7 is hydrolyzed preferentially, followed by position 4′ [11].

The methods for quantitative and qualitative analysis of KP methoxyflavones have been established [9]. After oral administration, the parameters of 5,7,3′,4′-tetramethoxyflavone (Compound-**3**) (50 mg/kg) pharmacokinetics, including maximum concentration (*C*_*max*_), the time of maximum blood drug concentration (*T*_*max*_), and half-life (*T*_*1/2*_), have been calculated as 0.79 *μ*g/mL, 190 min, and 273 min, respectively. Based on the area under the curve (AUC) of oral administration, Compound-**3** has an estimated bioavailability of 14.3% [12]. Using isotope-labeling method, the metabolites of Compound-**3** have been identified as 7-hydroxy-5,3′,4′-trimethoxyflavone, 5-hydroxy-7,3′,4′-trimethoxyflavone (Compound-**11**), 7-hydroxy-5,3′,4′-trimethoxyflavone sulfate, 3′-hydroxy-5,7,4′-trimethoxyflavone, and 4′-hydroxy-5,7,3′-trimethoxyflavone [13].

Till now, there is not enough scientific evidence to elucidate the optimal dose. Recommendation from Thai traditional medicine institute suggests the daily dose of KP is 1.2g. But administration with 1.35g of KP daily does not produce any adverse effects [1]. In addition, the powder of KP extract has been developed as a food ingredient on the market, which is standardized for containing not less than 2.5% of 5,7-dimethoxyflavone (Compound-**6**) and 10% of total methoxyflavones [2]. Acute and chronic toxicity study has been proved that oral administration of KP does not induce any abnormal changes in body weight and histology in various visceral organs [14, 15]. Toxicological study exhibits that the ethanol KP extract (at the doses of 60, 120, and 240 mg/kg for 60 days) does not induce significant changes in hemoglobin, white blood cells, or differential cell count. No any negative effects on renal and hepatic functions have been found at the tested doses [16]. Fitnox, a sports nutritional supplement, is a unique blend of KP methoxyflavones, pomegranate peel polyphenols, and* Moringa oleifera* leaf saponins. Subchronic toxicological study shows that administration of Fitnox (at the dose of 1000 mg/kg/day for 90 days) to rats exhibits no any drug-related toxicity or mortality in either sex and no significant changes between the control and Fitnox treated groups in all parameters at the hematological, biochemistry, and histological levels [17]. Another study for evaluating the toxicology of the ethanol KP extract (5, 50, and 500 mg/kg/day for 6 months) demonstrates no notable histological changes in all groups. The hematological parameters are also within the normal range in both sexes. But the body weight and the triglyceride levels at the 500 mg/kg dose rats group are lower, and the glucose and cholesterol levels are higher [15].

## 3. Cellular Metabolism-Regulating Activity

Obesity, closely associated with insulin resistance, is often caused by excessive calories intake and lack of exercise. The former promotes accumulation of triglycerides in the adipocyte lipid droplets and inhibition of lipolysis, which is regulated by adipose triglyceride lipase (ATGL), hormone-sensitive lipase (HSL), and monoacylglycerol lipase. The KP extracts, Compound-**1**, and Compound-**4** have been demonstrated to increase lipolysis, suppress lipid accumulation, and decrease hypertrophy in mature adipocytes through activation of ATGL and HSL expression in a peroxisome proliferator-activated receptor *γ* (PPAR*γ*)-independent manner. In contrast, quercetin, the full demethylation of Compound-**1**, does not show any suppressive activity in lipid accumulation in mature adipocytes under the same conditions. These suggest that the methoxyl groups in the effective methoxyflavones play a crucial role for this activity [4] (Table 1).

Consistently, in high-fat diet (HFD)-treated mice, the KP extracts significantly promote energy expenditure through activation of brown adipose tissue (BAT) and upregulation of uncoupling protein 1 (UCP1) expression, which is exclusively expressed in BAT. These lead to decrease of intra-abdominal fat accumulation, plasma triglyceride, and leptin levels [18, 19]. These data critically support the antiobesity effects of KP in Tsumura, Suzuki, Obese Diabetes (TSOD) mice, which is a mouse model of spontaneous obese type II diabetes [19]. In the process of adipogenesis, CCAAT/enhancer-binding protein *β* (C/EBP*β*), a member of C/EBP family, is induced by hormonal signals and functions as a positive transcriptional mediator of PPAR*γ*. On the other hand, the expression of PPAR*γ* is suppressed by GATA family proteins at the early stage of adipocyte differentiation. The ethyl acetate extract of KP, compound**-1**, and compound**-4** has been shown to upregulate C/EBP*β* and PPAR*γ* expression and downregulate GATA-2 expression, resulting in promotion of adipogenesis and secretion of adiponectin in 3T3-L1 preadipocytes. However, methoxyflavones isolated from KP have been tested and confirmed that they are not the ligands of PPAR*γ* [20].

Further investigation of the possible mechanism of KP in regulating energy metabolism shows that the ethyl acetate extract of KP suppresses the whitening of interscapular BAT in TSOD mice. These might be associated with upregulation of PPAR*γ*, UCP-1, and *β*3AR expression and accumulation of triacylglycerol in primary brown preadipocytes. These results suggest that the extract of KP may promote the differentiation of brown adipocytes and increase the thermogenesis effects [21]. In predifferentiated (p) and differentiated (d) C2C12 myoblasts, the KP extracts, Compound**-6**,** -12**, and** -15** significantly upregulate the expression of glucose transporter 4 (GLUT4) and monocarboxylate transporter 1 (MCT1) and PPAR*γ* coativator-1*α* (PGC-1*α*), leading to increased production of ATP and mitochondrial biogenesis [22].

Mitochondrial biogenesis in skeletal muscle may benefit from exercise by improving mitochondrial functions and the response to energy demands. The standardized extract of KP has been shown to increase running endurance and ratio of skeletal muscle weight/body weight. KP extract also upregulates the expression of PGC-1*α* and its upstream factors Sirt1, adenosine monophosphate (AMP)-activated protein kinase (AMPK), and PPAR*δ* in C57BL/6J mice. In L6 myotubes, KP extract significantly promotes mitochondrial biogenesis and density due to increased expression of PGC-1*α*, nuclear respiratory factor-1 (NRF-1), estrogen-related receptor-*α* (ERR*α*), and mitochondrial transcription factor A (Tfam) [23] (Table 1). Consequently, the extract of KP significantly improves physical fitness performance and muscular endurance through upregulation of PGC-1*α* and glucose synthase (GS) expression in C2C12 cells. Meanwhile, the inflammatory cytokines IL-6 and TNF*α* expression are also attenuated in C2C12 cells by the KP extract [2].

Medicinal herbs may induce the alternation of certain drug metabolism and, likewise, the metabolism of some active constituents from medicinal herbs may be altered by drugs; specifically those can influence the activity of cytochrome P450 enzymes. CYP family members including CYP1, CYP2, and CYP3 are the crucial factors in drug metabolism. The ethanol extract of KP has been shown to alter the enzymatic activities of CYP1A1, CYP1A2, CYP2B, and CYP2E1* in vitro*. In mice administrated with the KP extract at the dose of 250 mg/kg, the activity of CYP1A1 and CYP1A2 increases after short-term treatment, CYP2E1 increases after long-term treatment, CYP2B increases all the time-points of treatment, but CYP3A does not change [10]. KP extracts have been demonstrated to influence the metabolism of sildenafil, which is a PDE-5 inhibitor for treating erectile dysfunction. KP extracts significantly decreases* C*_*max*_, AUC, and* T*_*1/2*_ of sildenafil by 40-52%, 60-65%, and 32-54%, respectively, while the elimination rate constant (*K*_*e*_) of sildenafil is increased by 37-77% [59].

## 4. Anticancer Activity

Medicinal herbs are of importance and interest for a successful additional strategy to manage cancers. The ethanol extract of KP has been demonstrated to dose-dependently suppress cell growth, decrease cell viability, and induce apoptosis in HL-60 cells, as indicated by cellular morphological changes, phosphatidylserine externalization, mitochondrial trans-membrane potential (MTP) loss, and caspase-3 activation. These activities can be enhanced by administration of inhibitors of PI3K, AKT, and MEK. Similarly, the 95% ethanol extract of KP inhibits cell proliferation and viability and promotes cell apoptosis in human promonocytic leukemic U937 cell line, as shown by condensed nuclei and apoptotic bodies and decreased MTP [25] (Table 1). The methanol extract of KP and several methoxyflavones, including Compound**-3, -7, -10, **and** -11**, have been demonstrated to exhibit inhibitory activity on melanogenesis in theophylline-induced murine B16 melanoma 4A5 cells (IC_50_ = 9.6 *μ*g/mL, 8.6 *μ*M, 2.9 *μ*M, 3.5 *μ*M, and 8.8 *μ*M, respectively). Additionally, there is not any notable cytotoxicity of the isolated compounds on B16 melanoma 4A5 cells at the effective doses which is detected. The possible mechanism might be associated with the suppressive effects of these compounds against the expression of tyrosinase, tyrosine-related protein (TRP)-1, and TRP-2 [8] (Table 1).

In cervical cancer HeLa cells, the ethanol extract of KP promotes cell apoptosis through suppression of PI3K/AKT and MAPK signaling pathways. In addition, KP also inhibits the migration and invasion of HeLa cells. This might be related to the inhibitory effects on MMP-2 expression [26]. Evaluation of the ethanol extract of KP effects on human cholangiocarcinoma cell lines (HuCCA-1 and RMCCA-1) has been shown that the CC50s values on HuCCA-1 and RMCCA-1 are 46.1 *μ*g/mL and 62.0 *μ*g/mL, respectively [10]. P-glycoprotein (P-gp), a member of the ATP-binding cassette (ABC) family, extrudes various chemotherapeutic agents during cancer management, leading to multidrug resistance. The ethanol extract of KP and Compound**-1** have been found to increase the accumulation of rhodamine 123 and daunorubicin, the substrates of P-gp, in LLC-GA5-COL 150 cells, which is a transfectant cell line of a porcine kidney epithelial cell line LLC-PK1 with human* MDR1* cDNA [27]. The multidrug resistance associated proteins (MRPs) including MRP-1, -2, and -3 are also implicated in multidrug resistance. MRPs inhibitors can significantly induce the accumulation of calcerin, a MRPs substrate, which is not affected by the typical P-gp inhibitor verapamil. The ethanol extract of KP and the methoxyflavones have been found to increase the accumulation of calcein and doxorubicin in A549 cells dose-dependently by inhibiting MRPs functions. Compound**-6** shows the most potent stimulatory activity in accumulation of doxorubicin in A549 cells. However, it seems that 5-hydroxy-methoxyflavones are excluded for association with such stimulatory effects [28].

## 5. Vascular Relaxant and Cardioprotective Activity

The ethanol extract of KP has been shown to induce relaxation of aortic ring and ileum in phenylephrine (PE)- or acetylcholine (ACH)-induced isolated tissues dose-dependently [29]. However, with high hydrophobicity property, Compound**-6** may cause serious problems after injection. Complexation of Compound**-6** with 2-hydroxypropyl-*β*-cyclodextrin significantly increases the water solubility for 361.8-fold and the inhibitory effect on BChE for 2.7-fold [60].

NO, a signaling molecule, is produced by NO synthases, which have three isoforms, i.e., iNOS, eNOS, and nNOS. NO plays a critical role in maintaining normal vascular functions, and its expression is tightly controlled. NO generated by iNOS in a “high output” manner is involved in cell dysfunction and apoptosis. In contrast, NO produced by eNOS in a “low output” manner plays an important role in preventing cardiovascular diseases. The ethanol extract of KP has been shown to significantly upregulate the expression of eNOS and NO dose-dependently in HUVECs [30]. After 6 weeks of administration with the dichloromethane extract of KP to middle-aged male rats, contraction in thoracic aorta and mesenteric artery to phenylephrine is decreased and vasorelaxation to acetylcholine but not to glyceryl trinitrate is increased. The underlying mechanism might be possibly associated with upregulation of eNOS expression and NO production [31]. Similar results are also reached by Malakul (2011), who further demonstrated that the ethanol extract of KP significantly reduces the production of superoxide and improves endothelial dysfunction in STZ-induced aortic rings [32] (Table 1).

Ischemia and reperfusion (I/R) injury is often induced by accumulation of reactive oxygen species (ROS) and calcium in the cytosol and mitochondria. The ethanol extract of KP has been shown to exhibit cardioprotective effects on I/R injury through activation of cGMP-NO, attenuation of calcium influx, and defense against ROS in rat aorta rings [33]. Consistently, Compound**-1** significantly enhances the expression of eNOS and cystathionine-*γ*-lyase (CSE) and increases the productions of NO and H_2_S, leading to increased vasodilatation to acetylcholine and decreased contraction to phenylephrine in middle-age male rats. In addition, Compound**-1** lowers the concentration of plasma glucose but elevates the level of plasma high-density lipoprotein cholesterol (HDLC) [34].

## 6. Sexual Enhancing Activity

KP has long been used for men sexual enhancement in Thailand. The activities of KP ethanol extract in male rat sexual behavior and its toxicity have been determined. In the first 10-minute period, all groups but the high dose of KP ethanol extract group (240 mg/kg) increase courtship behavior greatly, compared with those in the second and third 10-minute periods. However, no significant difference has been observed in mount latency, mount frequency, intromission latency, and intromission frequency between the treated and the controlled group. KP has been demonstrated to be a health-promoting herb. Animal experimental studies do not show any obvious toxicity, as indicated by unaffected functions of liver and kidney. But it is inadvisable to use high and chronic doses of KP for human sexual activity [16]. To further investigate the aphrodisia property of KP, male rats models are established with streptozotocin (STZ)-induced diabetes, which is associated with the complication of sexual dysfunction. KP treatment significantly elevates the levels of serum testosterone, the concentration of sperm, and the weight of testes and improves the behavior of copulation in STZ-induced diabetic rats [39] (Table 1). Similar effects of KP extract in aging rats on enhancement of sexual activity, which might be associated with increased dopaminergic function in hypothalamus.

The testosterone-like effects of KP on reproduction have been examined and found that KP at the dose of 1000 mg/kg does not change the mating behavior, the weights of reproductive organs, and the levels of serum hormones, including follicle stimulating hormone (FSH), luteinizing hormone (LH), and testosterone [61, 62]. Similarly, the influence of KP extracts by different solvents, including ethanol, hexane, and water, on male rat reproductive organs and the sexual functions has been investigated. No changes on the reproductive organ weights, any sexual behavior parameters, fertility, or sperm motility in Sprague-Dawley (SD) rats are observed in hexane and water extracts. However, the ethanol extract, in a dose dependent manner, increases the blood flow to the testis significantly, indicating an aphrodisiac activity [40] (Table 1).

Impaired relaxation of the penile arteries or the corpus cavernosum induced by multifactorial causes is the main pathological mechanism of erectile dysfunction (ED). Phosphodiesterase-5 (PDE-5) is the key enzyme to regulate the level of cGMP, which induces relaxation and vasodilation of vascular smooth muscle and increases blood flow to penile tissue. It has been demonstrated that the extract of KP and its 7-methoxyflavones show inhibitory effects on PDE-5 using the two-step radioactive assay. Compound**-6** exhibits the most potent inhibitory activity on PDE5 with an IC_50_ value of 10.64±2.09 *μ*M [7].

## 7. Neuroprotective Activity

The impacts of KP on neuropharmacological and neuroprotective activities* in vivo* and* in vitro* have been investigated. With application of two-dimensional gel electrophoresis (2D-gel), the ethanol extract of KP upregulates 37 proteins expression and downregulates 14 proteins in the hippocampus of Sprague-Dawley (SD) rats, of which the expressions of GFAP and DPYSL2 were associated with antioxidative and microtubule-forming activities, respectively. They were validated by western-blot and shown to be upregulated significantly. However, it was surprise to find that phosphorylated DPYSL2 expression was downregulated. In addition, KP extract also increased the productions of norepinephrine (NE), serotonin (5-HT), and dopamine (DA) in rat hippocampus [42]. KP extract has been included for managing psychiatric disorder and cognitive enhancement. However, the precise mechanisms are still under investigation.

*β*-site amyloid precursor protein cleaving enzyme 1 (BACE1), a rate-limiting enzyme associated with the abnormal expression of *β*-amyloid peptides, has been implicated in Alzheimer's disease (AD) pathological development. Compound**-1**, Compound**-4**, and Compound**-6** have been shown to inhibit BACE1 activity (IC50= 5.98×10^−5^M, 3.69×10^−5^M, and 4.95×10^−5^M, respectively) significantly in a noncompetitive manner, without affecting the activities of *α*-secretase or other serine protease [43]. The ethanol extract of KP has been demonstrated to exhibit antidepressant-like activity in rats using forced swimming test. The KP extract can significantly protect from valproic acid (VPA)-induced cognitive decline and proliferating cells reduction. In addition, the expression of doublecortin (DCX) is upregulated in hippocampus by KP extract. These indicate that KP extract might improve spatial memory and cells proliferation damaged by VPA within subgranular zone [44] (Table 1). Consistently, KP extract shows neuroprotective activity against impaired learning and memory induced by chronic restraint stress, as indicated by increased neuron density in all the areas of the hippocampus [63].

## 8. Antiallergic, Anti-Inflammatory, and Antioxidative Activity

The effects of the* n*-hexane or the 50% ethanol extracts of KP on inhibiting degranulation in rat basophilic leukemia (RBL-2H3) cells triggered by an IgE antigen or a calcium ionophore have been investigated and shown to have positive effects. Five components from the* n*-hexane extract responsible for such activity are identified. They are Compound-**2**, Compound-**5**, Compound-**6**, Compound-**10**, and Compound-**14**, of which Compound-**6** and Compound-**10** show more activity in inhibiting degranulation and the expression of inflammatory mediators [45]. These data are consistent with Tewtrakul (2008) research work that Compound-**10** and Compound-**6** have been demonstrated as the main compounds responsible for antiallergic activity in inhibiting degranulation with an IC_50_ value of 8.0 *μ*M and 20.6 *μ*M, respectively. The possible mechanism might be associated with the suppression of Ca^2+^ influx to the cells [5].

Macrophages RAW264.7 cells are known to generate various proinflammatory mediators, such as prostaglandin E2 (PGE2), interleukin 1*β* (IL-1*β*), tumor necrosis factor *α* (TNF*α*), and nitric oxide (NO). The ethanol extract of KP has been reported to inhibit the expression of PGE2 in lipopolysaccharide (LPS)-induced RAW264.7 cells with an IC_50_ value of 9.2 *μ*g/mL.* In vivo*, the hexane and chloroform fractions of KP extract show greater activity in decrease of rat paw edema than ethyl acetate, ethanol, and water fractions [46]. Consistently, the compounds isolated from KP have been tested for inhibitory effects on NO, PGE2, and TNF*α* release. Compound-**10 **has been demonstrated the highest activity against LPS-induced NO release (IC_50_=16.1*μ*M) and PGE2 production (IC_50_=16.3*μ*M) in RAW264.7 cells, but it is inactive on TNF*α* expression (IC_50_ > 100*μ*M) [47]. Meanwhile, Compound-**4**, Compound-**6**, and Compound-**10** significantly suppress the expression of NO, iNOS, and TNF*α* in LPS-induced RAW264.7 cells through activation of spleen tyrosine kinase (SYK) pathway but not ERK and JNK pathways [46, 48].

The ethanol extract of KP has been shown to decrease oxidative stress in diabetes [64]. ROS generation by inflammatory response-induced low-density lipoproteins (LDLs) oxidation is one of the main risk factors for atherosclerosis development. The extract of KP can significantly decrease the levels of NO in LPS-triggered RAW264.7 cells, inhibit the adhesive activity of human monocytic leukemia (THP-1) cells to human umbilical vein endothelial cells (HUVECs), downregulate the expression of cell adhesion molecules (CAMs) and inflammatory cytokines, and decrease the production of angiotensin-converting enzyme (ACE)-mediated Ang-II, leading to amelioration of oxidative stress [49] (Table 1).

## 9. Antiosteoarthritis Activity

Osteoarthritis (OA) is a chronic inflammatory disease. Nonsteroidal anti-inflammatory drugs (NSAIDs) have been proved be effective for improving joint functions. The extract of KP has been shown to ameliorate the severity of cartilage lesions in monoiodoacetic acid (MIA)-induced rat OA models. In IL-1*β*-treated human chondrocytes, the extract of KP, Compound-**4**, and Compound-**6** have been found to downregulate the expression of matrix metalloproteinases (MMPs), which degrade collagen in articular cartilage [65]. Compound-**3**, also a main methoxyflavones found in* Murraya exotica*, has been demonstrated to decrease the levels of IL-1*β*, TNF*α*, and PGE2 in rat OA knee synovial fluid. In cultured rat chondrocytes, Compound-**3** significantly inhibits EP/cAMP/PKA signaling pathway and *β*-catenin signaling pathway, leading to articular cartilage and chondrocytes protection [50] (Table 1).

In addition, Compound-**3** also exhibits chondroprotective effects on attenuation of endoplasmic reticulum stress (ERS) induced by proinflammatory cytokines PGE_2_, as indicated by downregulation of PERK-CHOP signaling, IRE1-JNK signaling, ATF6 signaling, and GSK-3*β* expression and upregulation of GRP78 and XBP1 expression [51]. ERS promotes the expression of GSK-3*β*, which in turn triggers the activation of ERS. To investigate the underlying mechanism of ERS in inducing chondrocytes apoptosis, the knockout or overexpression of GSK-3*β* in transfected chondrocytes are established. GSK-3*β* can significantly exaggerate ERS-induced chondrocytes apoptosis. Compound-**3** ameliorates chondrocytes apoptosis by downregulation of GSK-3*β* and ERS [52]. At the early stage of ERS, IRE1*α* deficiency significantly induces ERS-induced chondrocytes apoptosis. Compound-**3** has been found to reversely upregulate the expression of IRE1*α*, XBP1s, and Bcl-2 and downregulate the expression of CHOP, p-JNK, and caspase-3 [53] (Table 1).

## 10. Antimicroorganism Activity

KP, the medicinal herb of Zingiberaceae family, has been included to screen for potential antiviral protease activity. The methanol extract of KP has been demonstrated to inhibit HIV-1 protease, hepatitis C virus (HCV) protease, and human cytomegalovirus (HCMV) protease. Several methoxyflavones isolated from KP have been shown to inhibit HIV-1 protease, of which Compound**-6** and Compound**-15** exhibit the most potent inhibitory activity with IC_50_ values of 19 *μ*M [66]. The inhibitory effects on avian influenza virus (H5N1) replication are also investigated. Both ethanol and water extracts of KP significantly upregulate the mRNA expression of TNF-*α* and IFN-*β* in MDCK cells [67]. In addition, Compound**-3** and Compound**-4** have been shown antiplasmodial effects against* Plasmodium falciparum* with IC_50_ values of 3.70 and 4.06 *μ*g/mL, respectively. Compound**-2** and Compound**-4** exhibit antifungal activity against* Candida albicans *with IC_50_ values of 39.71 and 17.61 *μ*g/mL, respectively, and show antimycobacterial activity with the minimum inhibitory concentration (MIC) of 200 and 50 *μ*g/mL, respectively [68].

## 11. Transdermal Permeable Activity

The physicochemical features of most methoxyflavones from KP, including low molecular weight, low log*P*, and low melting point, comply with transdermal delivery requirements. To avoid the first pass metabolism and increase the low bioavailability of KP, a suitable monolithic drug-in-adhesive patch of main components from KP has been evaluated. The unsaturated oleic acid exhibits the greatest enhancing activity on the permeation of methoxyflavones. The formulation including 15% of KP, 3% of oleic acid, and 3% of menthol has been revealed to enhance the permeation activity of methoxyflavones in rats. The pharmacokinetic study of total methoxyflavones* in vivo* shows that the* C*_*max*_ is 218.08 ng/mL with* T*_*max*_ at 8h [69]. Similarly, the dichloromethane extract of KP has been formulated in solid lipid nanoparticles and increased the transdermal permeability [70]. In TSOD mice, the extract of KP has been revealed to decrease the thickness of subcutaneous fat layer and the infiltration of adipocytes into the dermis. In addition, KP also prevents against UVB-induced denaturation of collagenous fibers in the skin [54].

## 12. Miscellaneous Section

Benign prostate hyperplasia (BPH) is often caused by hormone imbalance, due to the overwhelming social stress and inappropriate diet habits. Dihydrotestosterone is generated from testosterone by 5*α*-reductase (5*α*R), which is the key factor for development of BPH. It has been demonstrated that the extracts of KP, Compound-**1**, and Compound-**3** show significantly inhibitory activity on 5*α*R expression* in vitro*. Additionally, the extract of KP decreases the weights of seminal vesicles and prostate in rat BPH models [55] (Table 1).

Oral administration of the ethanol extract of KP (30, 60, and 120 mg/kg) has been shown to inhibit gastric ulcer induction by indomethacin, HCl/EtOH and water immersion restraint stress in rats. The KP extract does not have any effects on gastric volume, acidity, and pH output but preserve gastric wall mucus at doses of 60 and 120 mg/kg [56]. Xanthine oxidase (XOD) inhibitors are expected to ameliorate hyperuricemia, which can induce metabolic syndrome and trigger gout disease. The methanol extract of KP and its main active components Compound**-1** and Compound**-3** have been revealed to inhibit XOD activity. The IC_50_ values of Compound**-1** and Compound**-3** in inhibiting XOD activities are 0.9 mM and >4 mM, respectively [71].

Ultraviolet radiation B (UVB)-triggered ROS generation and matrix metalloproteinases (MMPs) expression lead to damage of extracellular matrix (ECM) integrity, tissue remodeling, skin functions impairment, and ultimately photoaging formation. The ethanol extract of KP has been shown to exhibit antiphotoaging effects, as evidence by decrease of wrinkle formation and collagen fibers loss. These might be associated with suppression of UVB-induced MMP-2, -3, -9, and -13 expression by KP extract through attenuation of c-Jun/c-Fos signaling pathway, leading to increased production of COL1A1, COL3A1, and COL7A1. In addition, IL-1*β*, COX-2, and NF-*κ*B signaling are also suppressed by KP extract treatment [57]. To further investigate the underlying mechanism, H_2_O_2_-induced cellular senescence* in vivo *and* in vitro* is studied. KP extract significantly increases mitochondrial biogenesis and functions through upregulation of PGC-1*α*, ERR*α*, NRF-1, and Tfam expression. KP extract also increases cell growth and inactivates senescence-related *β*-galactosidase through inhibition of cell cycle inhibitors (p16, p21, p53, and pRb) and upregulation of cell cycle activators (E2F1 and E2F2). H_2_O_2_-induced upregulation of PI3K/AKT signaling pathways is also attenuated by KP extract through activation of forkhead box O3a (FoxO3a) and mammalian target of rapamycin (mTOR) [58] (Table 1).

## 13. Clinical Prospective

A systematic review on clinical effects of KP has been shown for positive benefits, but it is inconclusive due to small studies included [1]. Modern research technologies have demonstrated that KP can suppress body weight gain, inhibit lipid accumulation, and prevent from pathological changes resulted by insulin resistance, fatty liver, and hypertension [72]. The weight gain may be obtained by the imbalance between energy expenditure and energy intake. BAT plays a crucial role in controlling the whole-body energy expenditure and body fatness. The ethanol extract of KP at the dose of 100 mg causes a significant increase in whole-body energy expenditure by recruiting BAT in male volunteers aged 21-29 in Japan [24] (Table 1).

In Mong hill tribe in Thailand, KP is believed to enhance physical work capacity and reduce perceived efforts. KP extract at the doses of 25 mg or 90 mg for 8 weeks has been demonstrated to increase physical fitness performance in 30-second chair stand test and 6 min walk test, increase the scavenger enzymes (superoxide dismutase (SOD), catalase (CAT), and glutathione peroxidase (GSH-Px)) expression, and decrease malondialdehyde (MDA) production [73]. In the double-blind, placebo-controlled clinical trial, oral administration of Fitnox at the single dose of 250 mg can significantly increase the levels of NO_2_^−^ and NO_3_^−^ in serum and saliva, leading to enhancement of overall performance and physical endurance [74]. Consistently, KP extract has been found to improve physical fitness, as indicated by enhancement of grip and leg strength, balance, endurance, and locomotor activity. In addition, the daily visual analog scale (VAS) figure score, postphysical fitness test (PFT) VAS fatigue score, and chronic fatigue syndrome (CFS) score are found to be enhanced greatly than those in the placebo group [75].

However, a randomized, double-blind, and crossover study has been demonstrated that acute administration of KP (1.35g) does not enhance exercise performance, compared with the placebo, as confirmed by repeated sprint exercise and submaximal exercise to exhaustion in college males in Thailand [76]. In contrast, supplement with KP extract at 180 mg per day for 12 weeks, the soccer players are found to increase the right-hand trip strength and left-hand grip strength, compared with those in the placebo group. On the other hand, the back and leg strength, the 40-yard technical test, the sit-and reach test, the 50-metre sprint test, and the cardiorespiratory fitness test do not show any significant difference from those in the placebo group [77].

Blood circulation is closely associated with blood fluidity. It has been demonstrated that 70% methanol extract of KP significantly improves blood fluidity through activation of fibrinolysis, as indicated by elongation of euglobulin lysis time in disseminated intravascular coagulation (DIC) rat models and the fibrinolysis assays* in vitro*. Compound**-1**,** -3**,** -4**, and** -14** have been involved in activation of fibrinolysis [35]. In the ventricular fibrillation (VF) of swine heart model, the saline extract of KP at high doses of 100 mg/kg and 50 mg/kg is found to increase the defibrillation threshold (DFT) and the upper limit of vulnerability (ULV). But it does not change VF threshold. In addition, KP administration attenuates diastolic and systolic blood pressures [36] (Table 1). On the other hand, the extract of KP (100mg/kg) has been demonstrated to decrease cardiac functions in normal rat hearts through upregulation of cyclic guanosine monophosphate (cGMP) level and NO signaling and downregulation of Ca^2+^ transient [37]. This is consistent with Compound**-6-**induced vasorelaxation through increased K^+^ efflux and attenuated Ca^2+^ influx [38].

KP is believed to benefit men's sexual activity. However, the ethanol extract of KP at dose of 70 mg/kg does not have any effects on weights of reproductive organs but decreases mount latency, ejaculation latency, and postejaculation latency [78]. On the other hand, the ethanol extract of KP has been found to increase blood flow to the testis dose-dependently [40]. PDE-5 inhibition has become the strategy for management of ED. However, PDE-5 inhibitors require sexual stimulation to activate cGMP-NO and trigger erection. Thus, targeting for relaxation directly to corpus cavernosum might be a new effective approach for ED management. It has been demonstrated that Compound**-1** exhibits a relaxant activity on isolated human cavernosum precontrated by phenylephrine. The possible mechanism might be that Compound**-1** inhibits L-type Ca^2+^ channel and induces immobilization of Ca^2+^ from sarcoplasmic reticulum. On the other hand, Compound**-1** does not act as a K_Ca_ channel opener, a PDE inhibitor, and a Rho-kinase inhibitor but a rather weak stimulator of NO release [41]. KP extract has been demonstrated to potentially manage age-related erectile dysfunction. At the dose of 90 mg/day, KP extract significantly increases all parameters. However, it does not alter the concentration of testosterone, follicle stimulating hormone (FSH), and luteinizing hormone (LH) [79].

## 14. Concluding Marks

In this review, we focus on the biological activities of KP and its main effective methoxyflavones, including cellular metabolism-regulating activity, anticancer activity, vascular relaxation and cardioprotective activity, sexual enhancing activity, neuroprotective activity, antiallergic, anti-inflammatory, and antioxidative activity, antiosteoarthritis activity, antimicroorganism activity, and transdermal permeable activity. Interestingly, current investigations report that KP administration does not show any obvious acute (13.33 g/kg) and chronic (5, 50, and 500 mg/kg/day for 6 months) toxicity or mortality, as evidenced by no alternation in hematology and histology. Studies indicate that KP (70 mg/kg/day for 4 weeks) does not effectively affect the reproductive organ but increases sexual motivation. In contrast, other investigations show KP (200 mg/kg for 2 weeks) might enhance men's sexual performance through increasing dopaminergic function in hypothalamus. Another mechanism includes inhibition (60-70%) of PDE-5 activity by KP at the dose of 50 *μ*M. These could be the promising strategy for ED management. However, it is inadvisable to use high and chronic doses of KP for human sexual activity. KP (25 or 90 mg for 8 weeks) has been used for enhancing physical work capacity, as evidenced by increased expression of scavenger enzymes and decreased expression of MDA. Potentially, KP is used for promoting health through, at least, regulating the balance of energy metabolism and improving cardiovascular functions. The underlying mechanisms of KP in benefiting human are not fully clear. More efforts are needed.

## Figures and Tables

**Table 1:** The biological activities of KP and its methoxyflavones.

Activities	Extracts/compounds	Models	Doses	Biological activities	Ref.
Metabolism regulation	Ethanol KP extract: modified starch (3:7) for powder. 600 mg KP powder suspended in 40 mL water	Male ddY mice, C2C12	45mg/kg/day, 10 *μ*g/mL	Improve physical fitness performance and muscular endurance, increase PGC-1*α* and GS expression.	[2]
	Methanol KP extract dissolved in DMSO	3T3-L1	3, 10, and 30 *μ*g/mL	Activate ATGL/HSL/PPAR*γ*; increase lipolysis; decrease hypertrophy.	[4]
	Compound**-1**, **-4 **dissolved in DMSO	3T3-L1	5, 15, and 30 *μ*g/mL		
	50% ethanol extract was freeze-dried for powder	C57BL6J mice	HFD containing 0.5% or 1% KP extract for 7 weeks	Increase UCP1 expression, activate BAT.	[18]
	KP rhizome was pulverized with a Wonder Blender for KP powder	TSOD mice	1% and 3% KP powder in powder feed MF	Suppress body weight increase, visceral fat accumulation, hypertension, hyperinsulinemia, glucose intolerance, peripheral neuropathy, lipid metabolism abnormalities, and insulin resistance.	[19]
	Ethyl acetate KP extract dissolved in DMSO	3T3-L1	1 and 3 *μ*g/mL	Upregulate C/EBP*β* and PPAR*γ* expression and downregulate GATA-2 expression.	[20]
	Compound**-1**, **-4 **dissolved in DMSO	3T3-L1	3, 10, and 30 *μ*M		
	Ethyl acetate KP extract was pulverized for KP powder	TSOD mice,	0.3% and 1% KP mix with MF for 8 weeks	Upregulate PPAR*γ*, UCP-1, and *β*3AR expression, accumulate triacylglycerol.	[21]
	Ethyl acetate KP extract	Brown adipocytes	1, 3, 10, and 30 *μ*g/mL		
	70% ethanol KP extract dissolved in DMSO	pC2C12 dC2C12	1, 3, and 10 *μ*g/mL	Upregulate the expression of GLUT4, MCT1, and PGC-1*α*	[22]
	Compound**-6**,**-12**,**-15 **dissolved in DMSO		1 and 10*μ*M		
	95% ethanol KP extract	C57BL/6J	200 mg/kg/day + HFD for 10 weeks	Increase PGC-1*α*, NRF-1, NRF-1, and Tfam expression	[23]
		L6 myotubes	1 and 10 *μ*g/mL	Upregulate Sirt1/AMPK/PPAR*δ*/ PGC-1*α*	
	95% ethanol KP extract was prepared by lyophilization	Male mice	250 mg/kg KP extract in carboxymethyl cellulose for 7, 14, 21 days	Increase CYP1A1, CYP1A2, CYP2B, and CYP2E1 activities, but CYP3A not change.	[10]
	60% ethanol KP extract mixed with dextrin for dried powder	volunteers	100 mg in a pullulan capsule	Increase whole-body energy expenditure.	[24]

Anti-cancer	95% ethanol KP extract was prepared for freeze-dried powder dissolved in DMSO	U937	20, 40, 60, 80, and 100 *μ*g/mL	Inhibit proliferation and viability, increase apoptosis	[25]
	Methanol extract was partitioned into EtOAc-soluble fraction and methanol fraction	B16 melanoma 4A5	1, 2.5, 5, and 10 *μ*g/mL	Inhibit melanogenesis with IC_50_ = 9.6 and 4.9 *μ*g/mL for methanol extract and EtOAc-soluble fraction	[8]
	Compound**-3**,** -7**, **-10**, and **-11**	B16 melanoma 4A5	3,10, and 30 *μ*M	Inhibit melanogenesis, suppress tyrosinase, TRP-1, and TRP-2 expression.	[8, 26]
	95% ethanol KP extract was prepared by lyophilization and dissolved in DMSO	HeLa	10, 50, and 100 *μ*g/mL	Suppress PI3K/AKT and MAPK signaling, inhibit migration and invasion, inhibit MMP-2 expression	
	Tincture, ethanol, or aqueous extract and Compound**-1 **dissolved in DMSO	LLC-GA5- COL 150	1, 5, 10, 50, and 100 *μ*g/mL	Increase the accumulation of rhodamine 123 and daunorubicin	[27]
	Tincture, ethanol, or aqueous extract and Compound**-1 **dissolved in DMSO	A549	0.3-100 *μ*g/mL	Accumulate calcein and doxorubicin	[28]
	Ethanol extract (no details in preparation)	aortic ring	10 and 100 *μ*g/mL	Antagonize PE- or ACH-induced contraction.	[29]

Vascular relaxation and cardioprotection	95% ethanol KP extract dried under vacuum was dissolved in DMSO	HUVEC	1 and 10 *μ*g/mL	Upregulate the expression of eNOS and NO	[30]
	Dichloromethane fraction of KP ethanol extract	middle-aged male rats	100 mg/kg twice a day for 6 weeks	Decrease contraction to phenylephrine, increase vasorelaxation to acetylcholine.	[31]
	95% ethanol KP extract was prepared by lyophilization suspended in 15% Tween-20	aortic ring	1, 10, and 100 *μ*g/mL	Reduce superoxide anion	[32]
	95% ethanol KP extract was prepared by lyophilization suspended in 15% Tween-20 Compound**-1 **suspended in mixture (Tween 80: carboxy-methylcellulose sodium salt: distilled water = 0.2: 0.2: 10)	aortic ring middle-aged male rats	1, 10, and 100 *μ*g/mL 22 mg/kg twice a day for 6 weeks	Upregulate cGMP-NO signaling, inhibit Ca^2+^ influx, and reduce ROS	[33]
				Upregulate eNOS and CSE expression and downregulate NO and H_2_S production. Low down plasma glucose and elevate HDLC levels.	[34]
	70% methanol extract was lyophilized to dried extract and was suspended in 50 % propylene glycol aqueous solution	DIC rats	200 and 500 mg/kg for 7 successive days	prolong euglobulin lysis time, activate fibrinolysis	[35]
	Saline extract was filtered with Whatman filtered paper NO.1	Swine heart	12.5, 25, 50 and 100 mg/kg	Increase DFT and ULV, attenuate diastolic and systolic blood pressures.	[36]
	Saline extract was filtered with Whatman filtered paper NO.1	normal rat heart	12.5 and 100 mg/kg	Upregulate cGMP-NO signaling and downregulate Ca^2+^ transient.	[37]
	Compound**-6 **dissolved in DMSO	aortic ring	10, 30, and 100 *μ*M	Induce vasorelaxation, activate NO-eGMP, increase K^+^ efflux, and attenuate Ca^2+^ influx.	[38]
	The water-soluble powder of KP suspended in Tween 80 (no details in preparation for dried powder)	STZ-induced diabetic rats	140, 280, and 420 mg/kg	Increase serum testosterone levels, sperm concentration, teste weight, and improve sexual performance.	[39]

Sexual enhancement	Ethanol, hexane, and water extract prepared with polyvinylpyrrolidone (PVP)	SD rats	10, 20, and 40 mg/kg for 5 weeks	Only ethanol extract increases the blood flow to the testis.	[40]
	Ethanol extract was evaporated under reduced pressure until dried dissolved in DMSO	PDE-5 from mouse lung	50 *μ*g/mL	Show 62.63% inhibitory effect (IC_50_=12.24*μ*g/mL)	[7]
	Compound**-6 **dissolved in DMSO	PDE-5 from mouse lung	10 *μ*M	IC_50_ = 10.64±2.09 *μ*M	[7]
	Compound**-1**	Isolated human cavernosum	0.1 and 0.3 mM	Inhibit L-type Ca^2+^ channel, induce immobilization of Ca^2+^.	[41]
	Compound**-4**, **-6**	AChE, BChE	0.1 mg/mL	Inhibit the activity of AChE and BChE	[6]

Neuroprotection	Ethanol extract was dissolved in 2% Tween 80	SD rats	200 mg/kg	Increase NE, 5-HT, and DA production, increase GFAP and DPYSL2 expression.	[42]
	Compound**-1**, **-4**, **-6**	Recombinant human BACE1	3, 30, 50, and 100 *μ*g/mL	inhibit BACE1 activity (IC50= 5.98×10-5M, 3.69×10-5M, and 4.95×10-5M, respectively)	[43]
	95% ethanol KP extract dissolved in 0.5% carboxymethycellulose	Rats	100 mg/kg	Improve spatial memory and cells proliferation damaged by VPA.	[44]
	*n*-hexane extract was dried by nitrogen gas and dissolved in DMSO	RBL-2H3	125, 250, 500 *μ*g/mL	Inhibit degranulation; Inhibit the productions of TNF*α*, IL-4, and MCP-1.	[45]

anti-allergy anti-inflammation anti-oxidation	Compound**-6**, **-10 **was dissolved in 50% ethanol	RBL-2H3	50*μ*M	Inhibit degranulation; Inhibit the productions of TNF*α*, IL-4, and MCP-1. Inhibit PGE2 expression with IC_50_ value of 9.2 *μ*g/mL.	[45, 46]
	Ethanol extract was partitioned by hexane, chloroform, ethyl acetate, and water fractions and evaporated to dryness in vacuo	RAW264.7	3, 10, 30, and 100 *μ*g/mL		
	Compound**-10**	RAW264.7	3, 10, 30, and 100 *μ*M	Inhibit PGE2 and iNOS expression.	[46]
	Compound**-10 **dissolved in DMSO	RAW264.7	3, 10, 30, and 100 *μ*g/mL	Inhibit the release of NO (IC_50_=16.1*μ*M) and PGE2 (IC_50_=16.3*μ*M), but inactive on TNF*α* release.	[47]
	Chloroform fraction and ethanol extract were evaporated to dryness in vacuo	RAW264.7	1,3, 10, 30, and 100 *μ*g/mL	Inhibit NO release with IC_50_ value of 8.4 and 8.1 *μ*g/mL, respectively.	[48]
	Compound**-3**,**-4**,**-6**	RAW264.7	3, 10, and 30 *μ*g/mL	Inhibit NO release with IC_50_ value of 5.1, 4.6, and 8.7 *μ*g/mL, respectively. Suppress iNOS and TNF*α* expression through SYK pathway, but not ERK and JNK pathways.	[48]
	50% ethanol extract	RAW264.7, THP-1	250, 500, and 1000 *μ*g/mL	Decrease NO levels, inhibit THP-1 adhesive activity and CAMs and inflammatory cytokines expression, and ameliorate oxidative stress.	[49]
	Compound**-3**	Rat chondrocytes	5, 10, and 20 *μ*g/mL	Decrease IL-1*β*, TNF*α*, and PGE2 levels, inhibit EP/cAMP/PKA signaling and *β*-catenin signaling, downregulate PERK-CHOP signaling, IRE1-JNK signaling, and GSK-3*β* expression, upregulate GRP78 and XBP1 expression.	[50–53]

Anti-osteoarthritis	Ethanol extract	TSOD mice	1% KP mix with MF for 12 weeks	Decrease the thickness of subcutaneous fat layer and the infiltration of adipocytes into the dermis. Prevent against UVB-induced denaturation of collagenous fibers.	[54]

Transdermal permeation	Ethanol extract	Epididymis, BPH rat model	100, 200, 500 *μ*g/mL	Inhibit 5*α*R activity, decrease the weights of seminal vesicles and prostate.	[55]

Miscellaneous section	Compound**-1**, **-4**	Epididymis	50 *μ*M	Inhibit 5*α*R activity with IC_50_ values of 46.6 *μ*M and 48.7 *μ*M, respectively.	[55, 56]
	70% methanol KP extract was prepared by lyophilization and dissolved in propylene glycol	Rat	30, 60, and 100 mg/kg	Inhibit gastric ulcer induction, preserve gastric wall mucus, and exhibit no effects on gastric volume, acidity, and pH output.	
	95% ethanol extract was concentrated under reduced pressure	5-week-old female hairless mice	100 and 200 mg/kg	Suppress UVB-induced MMP-2, -3, -9, -13 IL-1*β*, COX-2, NF-*κ*B and c-Jun/c-Fos signaling.	[57]
	95% ethanol extract was concentrated under reduced pressure	8-week-old female hairless mice	200mg/kg/day for 24 weeks;	Increase PGC-1*α*, ERR*α*, NRF-1, Tfam, E2F1, E2F2, FoxO3a, and mTOR expression; inhibit *β*-galactosidase, p16, p21, p53, pRb, and PI3K/AKT expression.	[58]
	95% ethanol extract was concentrated under reduced pressure	Hs68 cells	1, 5, and 10 *μ*g/mL	Increase PGC-1*α*, ERR*α*, NRF-1, Tfam, E2F1, E2F2, FoxO3a, and mTOR expression; inhibit *β*-galactosidase, p16, p21, p53, pRb, and PI3K/AKT expression.	[58]
	

## References

[1] Saokaew S., Wilairat P., Raktanyakan P. (2017). Clinical Effects of Krachaidum (Kaempferia parviflora): A Systematic Review. *Evidence-Based Complementary and Alternative Medicine*.

[2] Toda K., Hitoe S., Takeda S., Shimoda H. (2016). Black ginger extract increases physical fitness performance and muscular endurance by improving inflammation and energy metabolism. *Heliyon*.

[3] Azuma T., Tanaka Y., Kikuzaki H. (2008). Phenolic glycosides from Kaempferia parviflora. *Phytochemistry*.

[4] Okabe Y., Shimada T., Horikawa T. (2014). Suppression of adipocyte hypertrophy by polymethoxyflavonoids isolated from Kaempferia parviflora. *Phytomedicine*.

[5] Tewtrakul S., Subhadhirasakul S., Kummee S. (2008). Anti-allergic activity of compounds from Kaempferia parviflora. *Journal of Ethnopharmacology*.

[6] Sawasdee P., Sabphon C., Sitthiwongwanit D., Kokpol U. (2009). Anticholinesterase activity of 7-methoxyflavones isolated from Kaempferia parviflora. *Phytotherapy Research*.

[7] Temkitthawon P., Hinds T. R., Beavo J. A. (2011). Kaempferia parviflora, a plant used in traditional medicine to enhance sexual performance contains large amounts of low affinity PDE5 inhibitors. *Journal of Ethnopharmacology*.

[8] Ninomiya K., Matsumoto T., Chaipech S. (2016). Simultaneous quantitative analysis of 12 methoxyflavones with melanogenesis inhibitory activity from the rhizomes of Kaempferia parviflora. *Journal of Natural Medicines*.

[9] Sutthanut K., Sripanidkulchai B., Yenjai C., Jay M. (2007). Simultaneous identification and quantitation of 11 flavonoid constituents in *Kaempferia parviflora* by gas chromatography. *Journal of Chromatography A*.

[10] Mekjaruskul C., Jay M., Sripanidkulchai B. (2012). Modulatory effects of Kaempferia parviflora extract on mouse hepatic cytochrome P450 enzymes. *Journal of Ethnopharmacology*.

[11] Kim M., Kim N., Han J. (2014). Metabolism of Kaempferia parviflora polymethoxyflavones by human intestinal bacterium Bautia sp. MRG-PMF1. *Journal of Agricultural and Food Chemistry*.

[12] Wei G.-J., Hwang L. S., Tsai C.-L. (2014). Absolute bioavailability, pharmacokinetics and excretion of 5,7,3',4'-tetramethoxyflavone in rats. *Journal of Functional Foods*.

[13] Lu W.-C., Sheen J.-F., Hwang L. S., Wei G.-J. (2012). Identification of 5,7,3′,4′-tetramethoxyflavone metabolites in rat urine by the isotope-labeling method and ultrahigh-performance liquid chromatography-electrospray ionization-mass spectrometry. *Journal of Agricultural and Food Chemistry*.

[14] Zhang M., Pan D. R., Zhou F. (2011). BP neural network extraction process by orthogonal beautiful azalea flavonoids. *Journal of Xinyang Normal University*.

[15] Chivapat S., Chavalittumrong P., Attawish A., Rungsipipat A. (2010). Chronic toxicity study of Kaempferia parviflora wall ex. Extract. *Thai Journal of Veterinary Medicine*.

[16] Sudwan P., Saenphet K., Saenphet S., Suwansirikul S. (2006). Effect of Kaempferia parviflora Wall. ex. Baker on sexual activity of male rats and its toxicity.. *The Southeast Asian Journal of Tropical Medicine and Public Health*.

[17] Jacob J., Amalraj A., Divya C., Janadri S., Manjunatha P., Gopi S. (2018). Oral toxicity study of sports nutritional powder in Wistar rats: A 90 day repeated dose study. *Toxicology Reports*.

[18] Yoshino S., Kim M., Awa R., Kuwahara H., Kano Y., Kawada T. (2014). Kaempferia parviflora extract increases energy consumption through activation of BAT in mice. *Food Science & Nutrition*.

[19] Akase T., Shimada T., Terabayashi S., Ikeya Y., Sanada H., Aburada M. (2011). Antiobesity effects of Kaempferia parviflora in spontaneously obese type II diabetic mice. *Journal of Natural Medicines*.

[20] Horikawa T., Shimada T., Okabe Y. (2012). Polymethoxyflavonoids from Kaempferia parviflora induce adipogenesis on 3T3-L1 preadipocytes by regulating transcription factors at an early stage of differentiation. *Biological & Pharmaceutical Bulletin*.

[21] Kobayashi H., Horiguchi-Babamoto E., Suzuki M. (2016). Effects of ethyl acetate extract of Kaempferia parviflora on brown adipose tissue. *Journal of Natural Medicines*.

[22] Toda K., Takeda S., Hitoe S., Nakamura S., Matsuda H., Shimoda H. (2016). Enhancement of energy production by black ginger extract containing polymethoxy flavonoids in myocytes through improving glucose, lactic acid and lipid metabolism. *Journal of Natural Medicines*.

[23] Kim M. B., Kim T., Kim C., Hwang J. K. (2018). Standardized Kaempferia parviflora Extract Enhances Exercise Performance Through Activation of Mitochondrial Biogenesis. *Journal of Medicinal Food*.

[24] Matsushita M., Yoneshiro T., Aita S. (2015). Kaempferia parviflora extract increases whole-body energy expenditure in humans: Roles of brown adipose tissue. *Journal of Nutritional Science and Vitaminology*.

[25] Banjerdpongchai R., Chanwikruy Y., Rattanapanone V., Sripanidkulchai B. (2009). Induction of apoptosis in the human leukemic u937 cell line by kaempferia parviflora wall.ex.baker extract and effects of paclitaxel and camptothecin. *Asian Pacific Journal of Cancer Prevention*.

[26] Potikanond S., Sookkhee S., Takuathung M. N. (2017). Kaempferia parviflora extract exhibits anti-cancer activity against HeLa cervical cancer cells. *Frontiers in Pharmacology*.

[27] Patanasethanont D., Nagai J., Yumoto R. (2007). Effects of Kaempferia parviflora extracts and their flavone constituents on p-glycoprotein function. *Journal of Pharmaceutical Sciences*.

[28] Patanasethanont D., Nagai J., Matsuura C. (2007). Modulation of function of multidrug resistance associated-proteins by Kaempferia parviflora extracts and their components. *European Journal of Pharmacology*.

[29] Wattanapitayakul S. K., Chularojmontri L., Herunsalee A., Charuchongkolwongse S., Chansuvanich N. (2008). Vasorelaxation and antispasmodic effects of Kaempferia parviflora ethanolic extract in isolated rat organ studies. *Fitoterapia*.

[30] Wattanapitayakul S. K., Suwatronnakorn M., Chularojmontri L. (2007). Kaempferia parviflora ethanolic extract promoted nitric oxide production in human umbilical vein endothelial cells. *Journal of Ethnopharmacology*.

[31] Yorsin S., Kanokwiroon K., Radenahmad N., Jansakul C. (2014). Effects of Kaempferia parviflora rhizomes dichloromethane extract on vascular functions in middle-aged male rat. *Journal of Ethnopharmacology*.

[32] Malakul W., Thirawarapan S., Ingkaninan K., Sawasdee P. (2011). Effects of Kaempferia parviflora Wall. Ex Baker on endothelial dysfunction in streptozotocin-induced diabetic rats. *Journal of Ethnopharmacology*.

[33] Malakul W., Ingkaninan K., Sawasdee P., Woodman O. L. (2011). The ethanolic extract of Kaempferia parviflora reduces ischaemic injury in rat isolated hearts. *Journal of Ethnopharmacology*.

[34] Yorsin S., Kanokwiroon K., Radenahmad N., Jansakul C. (2016). Increased vascular eNOS and cystathionine-*γ*-lyase protein after 6 weeks oral administration of 3, 5, 7, 3′, 4′-pentamethoxyflavone to middle-aged male rats. *Naunyn-Schmiedeberg's Archives of Pharmacology*.

[35] Murata K., Deguchi T., Fujita T., Matsuda H. (2013). Improvement in blood fluidity by Kaempferia parviflora rhizome. *Journal of Natural Medicines*.

[36] Weerateerangkul P., Surinkaew S., Chattipakorn S. C., Chattipakorn N. (2013). Effects of Kaempferia parviflora Wall. Ex. baker on electrophysiology of the swine hearts. *Indian Journal of Medical Research*.

[37] Weerateerangkul P., Palee S., Chinda K., Chattipakorn S. C., Chattipakorn N. (2012). Effects of Kaempferia parviflora Wall. Ex. baker and sildenafil citrate on cGMP level, cardiac function, and intracellular Ca2+ regulation in rat hearts. *Journal of Cardiovascular Pharmacology*.

[38] Tep-Areenan P., Sawasdee P., Randall M. (2010). Possible mechanisms of vasorelaxation for 5,7-dimethoxyflavone from Kaempferia parviflora in the rat aorta. *Phytotherapy Research*.

[39] Lert-Amornpat T., Maketon C., Fungfuang W. (2017). Effect of Kaempferia parviflora on sexual performance in streptozotocin-induced diabetic male rats. *Andrologia*.

[40] Chaturapanich G., Chaiyakul S., Verawatnapakul V., Pholpramool C. (2008). Effects of *Kaempferia parviflora* extracts on reproductive parameters and spermatic blood flow in male rats. *Reproduction*.

[41] Jansakul C., Tachanaparuksa K., Mulvany M. J., Sukpondma Y. (2012). Relaxant mechanisms of 3, 5, 7, 3′, 4′-pentamethoxyflavone on isolated human cavernosum. *European Journal of Pharmacology*.

[42] Plaingam W., Sangsuthum S., Angkhasirisap W., Tencomnao T. (2017). Kaempferia parviflora rhizome extract and Myristica fragrans volatile oil increase the levels of monoamine neurotransmitters and impact the proteomic profiles in the rat hippocampus: Mechanistic insights into their neuroprotective effects. *Journal of Traditional and Complementary Medicine*.

[43] Youn K., Lee J., Ho C.-T., Jun M. (2016). Discovery of polymethoxyflavones from black ginger (Kaempferia parviflora) as potential *β*-secretase (BACE1) inhibitors. *Journal of Functional Foods*.

[44] Welbat J. U., Chaisawang P., Chaijaroonkhanarak W. (2016). Kaempferia parviflora extract ameliorates the cognitive impairments and the reduction in cell proliferation induced by valproic acid treatment in rats. *Annals of Anatomy*.

[45] Horigome S., Yoshida I., Tsuda A. (2014). Identification and evaluation of anti-inflammatory compounds from Kaempferia parviflora. *Bioscience, Biotechnology, and Biochemistry*.

[46] Sae-wong C., Tansakul P., Tewtrakul S. (2009). Anti-inflammatory mechanism of Kaempferia parviflora in murine macrophage cells (RAW 264.7) and in experimental animals. *Journal of Ethnopharmacology*.

[47] Tewtrakul S., Subhadhirasakul S. (2008). Effects of compounds from Kaempferia parviflora on nitric oxide, prostaglandin E2 and tumor necrosis factor-alpha productions in RAW264.7 macrophage cells. *Journal of Ethnopharmacology*.

[48] Sae-Wong C., Matsuda H., Tewtrakul S. (2011). Suppressive effects of methoxyflavonoids isolated from Kaempferia parviflora on inducible nitric oxide synthase (iNOS) expression in RAW 264.7 cells. *Journal of Ethnopharmacology*.

[49] Horigome S., Yoshida I., Ito S. (2017). Inhibitory effects of Kaempferia parviflora extract on monocyte adhesion and cellular reactive oxygen species production in human umbilical vein endothelial cells. *European Journal of Nutrition*.

[50] Wu L., Liu H., Li L. (2014). 5,7,3′,4′-Tetramethoxyflavone exhibits chondroprotective activity by targeting *β*-catenin signaling in vivo and in vitro. *Biochemical and Biophysical Research Communications*.

[51] Yang J., Liu H., Li L., Liu H. (2015). The Chondroprotective Role of TMF in PGE_2_-Induced Apoptosis Associating with Endoplasmic Reticulum Stress. *Evidence-Based Complementary and Alternative Medicine*.

[52] Yuan X., Li L., Shi W. (2017). TMF protects chondrocytes from ER stress-induced apoptosis by down-regulating. *Biomedicine pharmacotherapy = Biomedecine pharmacotherapie*.

[53] Wu L., Liu H., Li L. (2018). 5,7,3′,4′-Tetramethoxyflavone protects chondrocytes from ER stress-induced apoptosis through regulation of the IRE1*α* pathway. *Connective Tissue Research*.

[54] Hidaka M., Horikawa K., Akase T. (2017). Efficacy of Kaempferia parviflora in a mouse model of obesity-induced dermatopathy. *Journal of Natural Medicines*.

[55] Murata K., Hayashi H., Matsumura S., Matsuda H. (2013). Suppression of benign prostate hyperplasia by kaempferia parviflora rhizome. *Pharmacognosy Research*.

[56] Rujjanawate C., Kanjanapothi D., Amornlerdpison D., Pojanagaroon S. (2005). Anti-gastric ulcer effect of *Kaempferia parviflora*. *Journal of Ethnopharmacology*.

[57] Park J. E., Pyun H. B., Woo S. W., Jeong J. H., Hwang J. K. (2014). The protective effect of Kaempferia parviflora extract on UVB-induced skin photoaging in hairless mice. *Photodermatology, Photoimmunology & Photomedicine*.

[58] Park J. E., Woo S. W., Kim M. B., Kim C., Hwang J. K. (2017). Standardized* Kaempferia parviflora* Extract Inhibits Intrinsic Aging Process in Human Dermal Fibroblasts and Hairless Mice by Inhibiting Cellular Senescence and Mitochondrial Dysfunction. *Evidence-Based Complementary and Alternative Medicine*.

[59] Mekjaruskul C., Sripanidkulchai B. (2015). Pharmacokinetic interaction between Kaempferia parviflora extract and sildenafil in rats. *Journal of Natural Medicines*.

[60] Songngam S., Sukwattanasinitt M., Siralertmukul K., Sawasdee P. (2014). A 5,7-Dimethoxyflavone/hydroxypropyl-*β*-cyclodextrin inclusion complex with anti-butyrylcholinesterase activity. *AAPS PharmSciTech*.

[61] Trisomboon H., Tohei A., Malaivijitnond S., Watanabe G., Taya K. (2008). Oral administration of Kaempferia parviflora did not disturb male reproduction in rats. *The Journal of Reproduction and Development*.

[62] Trisomboon H., Watanabe G., Wetchasit P., Taya K. (2007). Effect of daily treatment with Thai herb, Kaempferia parviflora, in Hershberger assay using castrated immature rats. *The Journal of Reproduction and Development*.

[63] Wattanathorn J., Tong-Un T., Muchimapura S., Wannanon P., Sripanidkulchai B., Phachonpai W. (2013). Anti-stress effects of kaempferia parviflora in immobilization subjected rats. *American Journal of Pharmacology and Toxicology*.

[64] Kusirisin W., Srichairatanakool S., Lerttrakarnnon P. (2009). Antioxidative activity, polyphenolic content and anti-glycation effect of some Thai medicinal plants traditionally used in diabetic patients. *Medicinal Chemistry*.

[65] Kobayashi H., Suzuki R., Sato K. (2018). Effect of Kaempferia parviflora extract on knee osteoarthritis. *Journal of Natural Medicines*.

[66] Sookkongwaree K., Geitmann M., Roengsumran S., Petsom A., Danielson U. H. (2006). Inhibition of viral proteases by Zingiberaceae extracts and flavones isolated from *Kaempferia parviflora*. *Die Pharmazie*.

[67] Sornpet B., Potha T., Tragoolpua Y., Pringproa K. (2017). Antiviral activity of five Asian medicinal pant crude extracts against highly pathogenic H5N1 avian influenza virus. *Asian Pacific Journal of Tropical Medicine*.

[68] Yenjai C., Prasanphen K., Daodee S., Wongpanich V., Kittakoop P. (2004). Bioactive flavonoids from Kaempferia parviflora. *Fitoterapia*.

[69] Tuntiyasawasdikul S., Limpongsa E., Jaipakdee N., Sripanidkulchai B. (2015). A monolithic drug-in-adhesive patch of methoxyflavones from Kaempferia parviflora: In vitro and in vivo evaluation. *International Journal of Pharmaceutics*.

[70] Sutthanut K., Lu X., Jay M., Sripanidkulchai B. (2009). Solid lipid nanoparticles for topical administration of Kaempferia parviflora extracts. *Journal of Biomedical Nanotechnology*.

[71] Nakao K., Murata K., Deguchi T. (2011). Xanthine Oxidase Inhibitory Activities and Crystal Structures of Methoxyflavones from Kaempferia parviflora Rhizome. *Biological & Pharmaceutical Bulletin*.

[72] Shimada T., Horikawa T., Ikeya Y. (2011). Preventive effect of Kaempferia parviflora ethyl acetate extract and its major components polymethoxyflavonoid on metabolic diseases. *Fitoterapia*.

[73] Wattanathorn J., Muchimapura S., Tong-Un T. (2012). Positive Modulation Effect of 8-Week Consumption of *Kaempferia parviflora* on Health-Related Physical Fitness and Oxidative Status in Healthy Elderly Volunteers. *Evidence-Based Complementary and Alternative Medicine*.

[74] Jacob J., Gopi S., Divya C. (2018). A Randomized Single Dose Parallel Study on Enhancement of Nitric Oxide in Serum and Saliva with the Use of Natural Sports Supplement in Healthy Adults. *Journal of Dietary Supplements*.

[75] Toda K. (2016). Enhancement of physical fitness by black ginger extract rich in polymethoxyflavones: a double-blind randomized crossover trial. *Integrative Molecular Medicine*.

[76] Wasuntarawat C., Pengnet S., Walaikavinan N. (2010). No effect of acute ingestion of Thai ginseng (Kaempferia parviflora) on sprint and endurance exercise performance in humans. *Journal of Sports Sciences*.

[77] Promthep K., Eungpinichpong W., Sripanidkulchai B., Chatchawan U. (2015). Effect of Kaempferia parviflora Extract on Physical Fitness of Soccer Players: A Randomized Double-Blind Placebo-Controlled Trial. *Medical Science Monitor Basic Research*.

[78] Chaturapanich G., Chaiyakul S., Verawatnapakul V., Yimlamai T., Pholpramool C. (2012). Enhancement of aphrodisiac activity in male rats by ethanol extract of *Kaempferia parviflora* and exercise training. *Andrologia*.

[79] Wannanon P., Wattanathorn J., Tong-Un T. (2012). Efficacy assessment of Kaempferia Parviflora for the management of Erectile Dysfunction. *Online Journal of Biological Sciences*.

